# Artificial Endometrial Preparation for Frozen-Thawed Embryo Transfer with or without Pretreatment with Depot Gonadotropin Releasing Hormone Agonist in Women with Regular Menses 

**Published:** 2015-03

**Authors:** Elham Azimi Nekoo, Maryam Chamani, Ensieh Shahrokh Tehrani, Batool Hossein Rashidi, Fatemeh Davari Tanha, Vahid Kalantari

**Affiliations:** 1 Tehran University of Medical Sciences, Tehran, Iran; 2 Reproductive Health Research Center, Tehran University of Medical Sciences, Tehran, Iran; 3 Women’s hospital, Tehran University of Medical Sciences, Tehran, Iran; 4 St Maarten School of Medicine, American University of Integrative Sciences, Cole Bay, St. Maarten

**Keywords:** frozen-thawed embryo transfer; GnRH agonist; artificial cycle; endometrial preparation; pregnancy outcome

## Abstract

**Objective:** To investigate the reproductive outcome of artificial endometrial preparation with exogenous steroids for frozen-thawed embryo transfer with and without pre-treatment with depot gonadotropin releasing hormone agonist (GnRH-a) in women with regular menses.

**Materials and methods:** This is a prospective randomized clinical trial conducted in two ART centers on 176 women undergoing frozen-thawed embryo transfer. All patients received oral estradiol valerate 4 mg daily from day 2 to day 5 and 6 mg per day from day 6 to the day of the pregnancy test. In day 13 of cycle, an ultrasound examination was performed. After ultrasound confirmation of endometrial thickness (≥8 mm) and no ovarian activity, progesterone in cyclogest supp (800 mg daily) was added. The dose of estradiol would be increased to 8 mg per day if the endometrial thickness was less than 8mm. Two or 3 embryos were transferred via transcervical route 48 hours after the beginning of progesterone administration. In group A (93 patients), Difereline (3.75 mg Im), as a depot GnRH agonist was administered in the midluteal phase (day 21) of previous cycle. In the other group B (n = 83) steroid supplementation was commenced without prior pituitary suppression. Chemical and clinical pregnancy rates were compared in two groups.

**Results:** No significant differences were seen between two groups in terms of chemical pregnancy and clinical pregnancy rates.

**Conclusion:** The findings support the artificial protocol without any pretreatment suppressive drugs to reduce the adverse side effects of GnRH agonists also to minimize the costs.

## Introduction

The studies showed that cryopreservation is a method to increase cumulative pregnancy rate and reproduction rate, follicle recruitment determines IVF productivity rate via the number of embryo frozen and subsequent transfer. Freezing Embryo Transfer (FET) cycle is performed using different cycle regimens: Frozen/thawed embryos may be transferred into the uterus in a natural cycle, a hormone replacement cycle or a stimulated cycle. Transfer in natural cycle (without any drugs) is usually recommended in young women with regular menstrual cycles and ovulation. Hormone replacement cycle with or without GnRh agonists is usually recommended for older women, woman without ovaries or non-functioning ovaries, women with irregular infrequent menstrual cycles or ovulation. Different IVF clinics have different protocols for giving these medications and in some women GnRh agonists may be given in addition to hormone replacement to suppress any hormone production by the ovaries which may interfere with the treatment. Stimulated cycle is where fertility drugs such as gonadotropin injection is usually recommended for women do not ovulate regularly and did not respond to hormone replacement treatment in a previous cycle. There is insufficient evidence to recommend any one particular protocol for endometrial preparation over another with regard to pregnancy rates after embryo (1-4). This study designed to determine whether there is a difference in outcome between artificial cycle FET with and without pretreatment with GnRH agonists.

## Materials and methods

This is a prospective randomized clinical trial study which was approved by ethical committe of TUMS.The participiants are the infertile patients (male factor) aged 20 to 37 years who had regular menstrual cycles and previously undergone IVF or ICSI with the same induction protocol with embryo cryopreservation in Vali-e-Asr Infertility research center, Tehran University of Medical Sciences, and Shayamehr infertility clinic (Private outpatient), Tehran, Iran. The Patients who volunteered to participate signed informed consent and then accorging to computerized random allocation program were randomly divided into two groups with different endometrial preparation regimen for freezed embryo transfe from January 2010 to February 2011. In both groups, oral Estradiol Valerate was taken at 4 mg daily from day 2 to day 5, at 6 mg per day from day 6 to the day of the pregnancy test. In day 13 of cycle, an ultrasound examination was performed. After ultrasound confirmation of endometrial thickness (≥8 mm) and if a periovulatory follicle was not present at the day 13 ultrasound, progesterone in cyclogest supp (400 mg /Bd) was added. The dose of estradiol would be increased to 8 mg per day if the endometrial thickness was less than 8mm.Two or 3 embryos were transferred via transcervical route 48 hours after the beginning of progesterone administration. In this study, cryopreservation was performed at the pronuclear stage and only frozen-thawed embryos with score = 8 were transferred. Hormonal treatment was continued at least until pregnancy test performed 15 days after transfer. If the pregnancy occurred the estradiol valerate at the last dose and progesterone at 800 mg daily would be continued until approximately 12 weeks of gestation. In group A (93 patients), Difereline (3.75 mg Im), as a depot GnRH agonist was administered in the midluteal phase (day 21). In the other group B (n = 83) commenced steroid supplementation without prior pituitary desensitization. The data were analyzed with the SPSS version 16. Data were compared using the t-test and the Chi-square test. A p-value of <0.05 was considered statistically significant. The primary outcome was implantation, chemical pregnancy and abortion.

## Results

In this study, there was no significant difference between the groups in term of baseline characteristics (Table 1). 

**Table 1:** Comparison of baseline characteristics in two groups

	GnRH-a	No GnRH-a	p value
**No of subjects**	**93**	**83**	
**Age (mean ± SD) (year)**	**35.9 ± 7.6**	**34.7 ± 7.6**	**0.212**
**Duration of infertility (mean ± SD) (year)**	**8.9 ± 6.1**	**8.7 ± 6.3**	**0.815**
**Endometrial thickness (mm)**	**8.4 ± 1.7**	**8.1 ± 1.3**	**0.084**
**Transferred embryo (mean ± SD) (n)**	**3.2 ± 0.8**	**3.1 ± 0.6**	**0.369**
**Type of infertility**			
** Primary **	**51(54.8%)**	**53(63.9%)**	
** Secondary**	**42(45.2%)**	**30(36.1%)**	**0.225**

**Table 2:** Pregnancy outcome of frozen-thawed embryo transfer in both groups

	GnRH-a (n = 93)	No GnRH-a (n=83)	p value
**Implantation rate [n (%)]**	**23 (24.7%)**	**30 (36.1%)**	**0.099**
**Chemical pregnancy rate [n (%)]**	**22 (23.7%)**	**28 (33.7%)**	**0.139**
**Abortion rate [n (%)]**	**2 (1.1%)**	**4 (3.6%)**	**0.259**
**Clinical pregnancy [n (%)]**	**20 (22.6%)**	**24 (30.1%)**	

There was no significant difference in implantation, pregnancy and abortion rates between both groups too (Table 2).

## Discussion

The outcome of a Frozen-thawed embryo transfer Protocol depends on the exact synchronization between endometrial preparation and embryo development. Such preparation may be induced after artificial preparation of the endometrium with exogenous estrogen along with or without pituitary down regulation with GnRH agonist. In present study we compared endometrial preparation for Frozen-thawed embryo transfer with and without pituitary down regulation by GnRH down regulation. The results showed that no significant differences between two groups in terms of chemical and clinical pregnancy rate. 

In a study of Lee, no difference was found in terms of implantation and pregnancy rates in the FET cycles when a GnRH agonist or GnRH antagonist was used in the previous oocyte retrieval cycle (6). Our results suggest that GnRH antagonists do not have a detrimental effect on oocyte quality or embryo development. In this study GnRH agonist was used.

 In a natural FET cycle (where no medications are used before the embryo transfer), the cycle is tracked for ovulation using blood tests or ultrasound to measure the thickness and maturity of the endometrium (7). Frozen embryo transfer in women with regular menses must synchronize with ovulation in natural cycle or after artificially preparing the endometrium with estrogen and progestron. Hill et al in 2010 reported that the synthetic hormone protocol for frozen-thawed embryo cycles offers improved outcome when compared with a natural cycle protocol for frozen-thawed blastocyst-stage ET cycles (8). However results showed that there is no significant differences in term of implantation, chemical and clinical pregnancy.

Authors believe that in artificial endometrial preparation cycles without GnRH agonist use, estrogen should be used at day1-2 of menstrual cycle, after this time the inhibition of spontaneous ovulation questionable. They suggested step-up protocol estradiol instead of fixed high dose estrogen because the former protocol is more similar to physiologic pattern and it is more suitable for endometrial maturation and then embryo implantation (8). In present study we used step-up protocol in both group for endometrial preparation.

It is believed that endometrial receptivity needs endometrial thickness between 5-8mm.Lower and shorter dose protocol results to more abortion rate (9). An optimal endometrial proliferation is necessary for optimal development of progesterone receptors and transformation to endometrium which is suitable for implantation (9).

Others believe that endometrial preparation for blastocyst -FET cycle without pituitary down regulation had similar pregnancy rate in the presence or absence of a dominant follicle (10). So they suggested that appearance of a dominant follicle couldn't predict the outcome of FET cycle in the protocol of endometrial preparation without pituitary down regulation. 

The studies suggested that in artificial endometrium preparation protocol without pituitary down-regulation, ultrasound examination of endometer can help to predict the outcome of cycle and evaluation of E2 level on progesterone initial day don’t give any other useful information (9). In this study only ulstrasound was used.

Some investigators suggested that pretreatment with GnRHa is benefit to avoid spontaneous ovulation and cycle cancelation. But many studies compared the two artificial protocols when using GnRH agonist or not and showed the similar reproductive outcome (10-12). The result of this study suggested that both FET protocols with and without pretreatment with GnRHa are equally effective in terms of implantation rate and pregnancy outcome in women with regular menstrual cycles. Also several studies used different kind of GnRHa but no evidence of statistically significant benefit was found for one GnRH agonist over another with regard to pregnancy rates after embryo transfers (11-14). In this study depot GnRH agonist administered. 

In conclusion, artificial endometrial preparation for FET with pretreatment with a GnRH agonist appears to be as effective as the artificial protocol without any pituitary suppression.

These findings support of the artificial protocol without any pretreatment suppressive drugs to reduce the adverse side effects of GnRH agonists also to minimize costs.
